# Extravascular MDCT Findings of Pulmonary Vein Stenosis in Children with Cardiac Septal Defect

**DOI:** 10.3390/children8080667

**Published:** 2021-07-30

**Authors:** Edward Y. Lee, Ryan Callahan, Sara O. Vargas, Kathy J. Jenkins, Halley J. Park, Zachary Gauthier, Abbey J. Winant

**Affiliations:** 1 Department of Radiology, Boston Children’s Hospital, Harvard Medical School, 300 Longwood Avenue, Boston, MA 02115, USA; Halley.Park@childrens.harvard.edu (H.J.P.); Abbey.Winant@childrens.harvard.edu (A.J.W.); 2 Department of Cardiology, Boston Children’s Hospital, Harvard Medical School, 300 Longwood Avenue, Boston, MA 02115, USA; Ryan.callahan@cardio.chboston.org (R.C.); Kathy.Jenkins@childrens.harvard.edu (K.J.J.); Zachary.Gauthier@childrens.harvard.edu (Z.G.); 3 Department of Pathology, Boston Children’s Hospital, Harvard Medical School, 300 Longwood Avenue, Boston, MA 02115, USA; Sara.Vargas@childrens.harvard.edu

**Keywords:** pulmonary vein stenosis, cardiac septal defect, multidetector computed tomography (MDCT) angiography, extravascular findings, children, pediatric patients

## Abstract

**Purpose**: To retrospectively investigate the extravascular thoracic MDCT angiography findings of pulmonary vein stenosis (PVS) in children with a cardiac septal defect. **Materials and Methods:** Pediatric patients (age ≤ 18 years) with cardiac septal defect and PVS, confirmed by echocardiogram and/or conventional angiography, who underwent thoracic MDCT angiography studies from April 2009 to April 2021 were included. Two pediatric radiologists independently evaluated thoracic MDCT angiography studies for the presence of extravascular thoracic abnormalities in: (1) lung and airway (ground-glass opacity (GGO), consolidation, pulmonary nodule, mass, cyst, septal thickening, fibrosis, and bronchiectasis); (2) pleura (pleural thickening, pleural effusion, and pneumothorax); and (3) mediastinum (mass and lymphadenopathy). Interobserver agreement between the two independent pediatric radiology reviewers was evaluated with kappa statistics. **Results:** The final study group consisted of 20 thoracic MDCT angiography studies from 20 consecutive individual pediatric patients (13 males (65%) and 7 females (35%); mean age: 7.5 months; SD: 12.7; range: 2 days to 7 months) with cardiac septal defect and PVS. The characteristic extravascular thoracic MDCT angiography findings were GGO (18/20; 90%), septal thickening (9/20; 45%), pleural thickening (16/20; 80%), and ill-defined, mildly heterogeneously enhancing, non-calcified soft tissue mass (9/20; 45%) following the contours of PVS in the mediastinum. There was a high interobserver kappa agreement between two independent reviewers for detecting extravascular abnormalities on thoracic MDCT angiography studies (k = 0.99). **Conclusion:** PVS in children with a cardiac septal defect has a characteristic extravascular thoracic MDCT angiography finding. In the lungs and pleura, GGO, septal thickening, and pleural thickening are frequently seen in children with cardiac septal defect and PVS. In the mediastinum, a mildly heterogeneously enhancing, non-calcified soft tissue mass in the distribution of PVS in the mediastinum is seen in close to half of the pediatric patients with cardiac septal defect and PVS.

## 1. Introduction

Pulmonary vein stenosis (PVS) is a rare and often challenging diagnosis, with an unfavorable prognosis, particularly in the pediatric population [1,2,3,4,5]. Characterized by a progressive reduction in the luminal size of one or more pulmonary veins, PVS presents with non-specific clinical signs and symptoms, such as failure to thrive, feeding intolerance, and shortness of breath, due to elevated pulmonary venous pressure [1,2,4]. Imaging evaluation, which can visualize the decreased caliber of the affected pulmonary veins, is essential for early and accurate diagnosis, as well as treatment strategy [6,7,8].

There are currently several imaging modalities available for evaluating pulmonary vein stenosis, including echocardiogram, conventional catheterization, multidetector computed tomography (MDCT), magnetic resonance imaging (MRI), and nuclear medicine study [6,7,8,9]. Among these modalities, MDCT offers high spatial resolution, fast scanning speed, and avoids the risks of sedation [10,11]. Consequently, MDCT has begun to assume an important role as a non-invasive imaging modality that simultaneously visualizes the pulmonary vein luminal caliber narrowing and provides comprehensive thoracic anatomic information in pediatric patients with PVS [6,7,8].

The characteristic vascular abnormalities of PVS on MDCT are well-known in both the pediatric and adult populations [6,7,8,12]. In addition, a recently published study showed characteristic extravascular MDCT abnormalities involving the lungs and mediastinum in children with primary PVS (i.e., without underlying congenital heart disease) [13]. However, to our knowledge, there is currently no published study that has investigated the extravascular thoracic MDCT angiography findings in children with PVS associated with isolated cardiac septal defects. Therefore, the goal of our study is to retrospectively investigate the extravascular thoracic MDCT angiography findings of PVS in children with a cardiac septal defect.

## 2. Methods

### 2.1. Institutional Review Board Approval

This retrospective study, which was obtained in compliance with the Health Insurance Portability and Accountability Act (HIPAA), was approved by our Institutional Review Board. Informed consent was waived due to the retrospective nature of this study. However, patient confidentiality was maintained in accordance with HIPAA guidelines.

### 2.2. Study Population

Our hospital’s radiology, cardiology, and pathology departmental electronic databases were used to collect all consecutive pediatric patients (≤18 years) with a known diagnosis of the underlying cardiac septal defect (i.e., ASD (atrial septal defect), VSD (ventricular septal defect), and CAVC (complete atrioventricular canal defect)) and secondary PVS, confirmed by echocardiogram and/or conventional angiography, who underwent thoracic MDCT angiography studies from April 2009 to April 2021. In addition, for each patient, demographic information, including age, gender, underlying cardiac anomalies, treatment (e.g., surgery and/or chemotherapy, transcatheter interventions (such as balloon angioplasty or stent placement)), and patient outcome was also collected.

There were 22 thoracic MDCT angiography studies from 22 individual pediatric patients with cardiac septal defect and PVS during the study period. Among them, 2 thoracic MDCT angiography studies were excluded due to substantial atelectasis in both lungs that markedly limited complete evaluation of lung parenchyma. Therefore, the final study group consisted of 20 diagnostic-quality thoracic MDCT angiography studies from 20 individual pediatric patients with cardiac septal defect and PVS. All 20 pediatric patients included in the final study group had only isolated cardiac septal defects, without a history of other congenital heart disease or medical conditions. ASD was seen in 7 patients (35%), CAVC was seen in 7 patients (35%), and VSD was seen in 6 patients (30%). For each patient, only the initial thoracic MDCT angiography study performed at the time of diagnosis of PVS was included. The mean time interval between the date of PVS diagnosis and thoracic MDCT angiography study was 2.7 months (SD: 4.9; range: 2 days to 7 months).

### 2.3. Pulmonary Vein Stenosis Diagnostic Criteria

Previously accepted criteria, including pulmonary vein luminal narrowing in ≥2 vessels with a mean gradient of ≥4 mm Hg seen on echocardiography or conventional angiography, were used to diagnose PVS [13,14]. Among the 20 pediatric patients included in the final study group, PVS was diagnosed based on echocardiography alone in 4 patients (20%), conventional angiography alone in 6 patients (30%), and both echocardiography and conventional angiography in 10 patients (50%).

### 2.4. Thoracic MDCT Angiography Technical Factors

#### 2.4.1. Types of MDCT Scanners

A total of 5 different MDCT scanners were used for 20 thoracic MDCT angiography studies in the final study group including: (1) a 16-MDCT scanner (*n* = 2; 10%); (2) a 64-MDCT scanner (*n* = 14; 70%); (3) a 96-MDCT scanner (*n* = 2; 10%); (4) a 128-MDCT scanner (*n* = 1; 5%); and (5) a 302-MDCT scanner (*n* = 1; 5%).

#### 2.4.2. Thoracic MDCT Technical Parameters

Optimized thoracic MDCT angiography parameters, using weight-based kilovoltage, low-dose tube current, and a high-speed mode (rotation time ≤ 1 s) closely following the ALARA (as low as reasonably achievable) principle, were used for all 20 thoracic MDCT angiography studies included in the final study group [15]. All 20 thoracic MDCT angiography studies were obtained with intravenous contrast. The intravenous contrast dose of 1.5–2 mL/kg was administered via mechanical injection, and CT scanning was initiated when optimal contrast enhancement (>200 Hounsfield unit (HU)) in the left atrium was observed on a monitoring scan by a dedicated pediatric CT technologist. CT scanning was performed in the cranial to caudal direction capturing the entire chest from the level of the thoracic inlet to the level of the diaphragm.

### 2.5. Thoracic MDCT Angiography Image Review

Axial and 2-dimensional multiplanar (e.g., sagittal and coronal) reformat MDCT images were generated in both standard lung (level, 500 Hounsfield units (HU); width, 1500 HU) and soft tissue (level, 40 HU; width, 450 HU) window settings at the CT console for review. A PACS (picture archiving and communication system) (Synapse, Fujifilm Medical Systems, Stamford, CT, USA) was used for MDCT image review.

To decrease potential reviewer bias, prior to the thoracic MDCT angiography image review process, any identifying information on the thoracic MDCT angiography images was removed. In addition, all reviewers were blinded to all other clinical and imaging study information. Furthermore, thoracic MDCT angiography studies were randomized prior to reviewing.

A total of 2 pediatric radiologists (pediatric thoracic radiologist and pediatric radiology fellow with 11 and 5 years of experience in interpreting pediatric thoracic MDCT angiography studies, respectively) independently reviewed all thoracic MDCT angiography studies. If there was a discrepancy between the findings of the two reviewers, a third radiologist, a pediatric thoracic radiologist with more than 20 years of experience in interpreting thoracic MDCT angiography studies, served as a tie-breaker and made the final decision. In order to minimize potential bias, this third reviewer was blinded to the two initial reviewer’s decisions on disagreed cases and all other clinical and imaging study information.

### 2.6. Thoracic MDCT Angiography Quality and Image Assessment

#### 2.6.1. Thoracic MDCT Angiography Quality Assessment

Prior to thoracic MDCT angiography image assessment, the quality of thoracic MDCT angiography studies was evaluated. The reviewers assessed each thoracic MDCT angiography study for the presence of motion artifact and degree of lung aeration [16]. The criteria for the presence of motion artifact were defined as the presence of one or more following MDCT findings including: (1) double-imaged structures; (2) blurring of the lung parenchymal interstitium or thoracic vessels; and (3) pulsation artifact. Substantial motion artifact and/or decreased lung aeration (>10% of the entire lung parenchymal area) were considered to be suboptimal and excluded from the final study group [16].

#### 2.6.2. Thoracic MDCT Angiography Image Assessment

Three main extravascular thoracic anatomic structures, including lung and airway, pleura, and mediastinum, were systematically evaluated based on previously established criteria [13,17,18]. The lung and airway were evaluated for the presence of: (1) ground-glass opacity (GGO); (2) consolidation; (3) nodule; (4) mass; (5) cyst; (6) septal thickening; (7) fibrosis; and (8) bronchiectasis [17]. The pleura was evaluated for the presence of pleural thickening, pleural effusion, and pneumothorax [18]. The mediastinum was evaluated for lymphadenopathy and masses. When a mediastinal mass was present, the location (in relation to the location of PVS), borders (well-circumscribed vs. ill-defined), contrast enhancement pattern (mild vs. avid and homogeneous vs. heterogeneous), and the presence of associated calcifications were also evaluated [13].

### 2.7. Statistical Analysis

Normally distributed variables were expressed as mean, standard deviation, and range. The number and percentage of abnormalities were calculated based on the proportion of abnormalities detected on thoracic MDCT angiography studies. Interobserver agreement between the two independent reviewers regarding thoracic MDCT angiography findings was measured by the Kappa statistics. As a guide for interpreting magnitudes for Kappa, we defined (low, moderate, high) agreement via the ranges (0.5–0.75, 0.75–0.9, 0.9–1). All statistical analysis was performed using SAS/STAT 14.1 [19].

## 3. Results

### 3.1. Study Population Characteristics

The final study group consisted of 20 thoracic MDCT angiography studies from 20 consecutive individual pediatric patients (13 males (65%) and 7 females (35%); mean age: 7.5 months; SD: 12.7; range: 2 days to 7 months) with cardiac septal defect and PVS. Among the 20 included patients, 12 patients (60%) underwent cardiac septal defect repair surgery prior to thoracic MDCT angiography studies, 6 patients (30%) underwent cardiac septal defect repair surgery after thoracic MDCT angiography studies, and the remaining 2 patients (10%) did not undergo cardiac septal defect repair surgery due to small VSD size. Presenting signs and symptoms in the 20 included patients were: pulmonary hypertension (*n* = 9), shortness of breath (*n* = 8), hypoxemia (*n* = 8), failure to thrive (*n* = 2), and bradycardia (*n* = 1).

### 3.2. Study Cohort Treatment and Outcome Information

In the final study population, 16/20 (80%) patients received chemotherapy for PVS, 10/20 (50%) patients were treated with transcatheter intervention (such as balloon angioplasty and/or stent placement), and 14/20 (70%) patients underwent surgery for the PVS. At the time of the last follow-up, 7/20 (30%) of patients in the study group were deceased due to complications of PVS.

### 3.3. Thoracic MDCT Angiography Quality Assessment Information

Based on the thoracic MDCT angiography quality assessment, there were 2 (9%) thoracic MDCT angiography studies with suboptimal quality (due to the substantial underlying atelectasis) out of 22 thoracic MDCT angiography studies from 22 individual pediatric patients during the study period. These two suboptimal quality thoracic MDCT angiography studies were excluded from the final study group. Therefore, the final study group consisted of 20 diagnostic-quality thoracic MDCT angiography studies from 20 individual pediatric patients with cardiac septal defect and PVS. There was no thoracic MDCT angiography study with substantial motion artifact.

### 3.4. Thoracic MDCT Angiography Findings

MDCT angiography study findings of extrathoracic abnormalities in the lung and airway, pleural, and mediastinal abnormalities in children with cardiac septal defect and PVS are detailed in Table 1.

**Figure 1:**
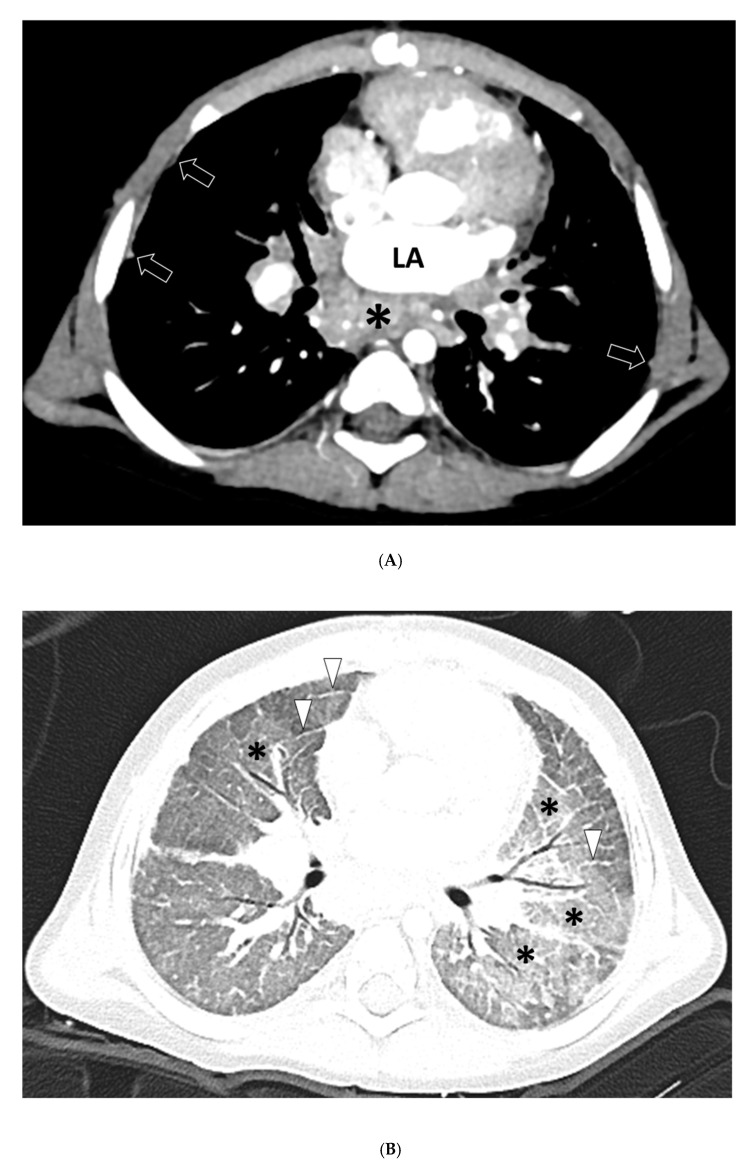
A 4-year-old male with complete atrioventricular canal defect and pulmonary vein stenosis who presented with pulmonary hypertension. (**A**) Axial contrast-enhanced thoracic MDCT angiography CT image shows ill-defined, mildly heterogeneously enhancing, non-calcified soft tissue mass (asterisk) in the expected location of bilateral pulmonary veins. Pleural thickening (arrow) is also seen. LA = Left atrium. (**B**) Axial lung window CT image demonstrates ground-glass opacities (asterisks) in both lungs and septal thickening (arrowheads).

Lung and airway abnormalities were seen in 18/20 (90%) thoracic MDCT angiography studies, including GGO (18/20; 90%) and septal thickening (9/20; 45%) (Figure 1). No consolidation, nodule, mass, cyst, fibrosis, or bronchiectasis was identified.

**Figure 2:**
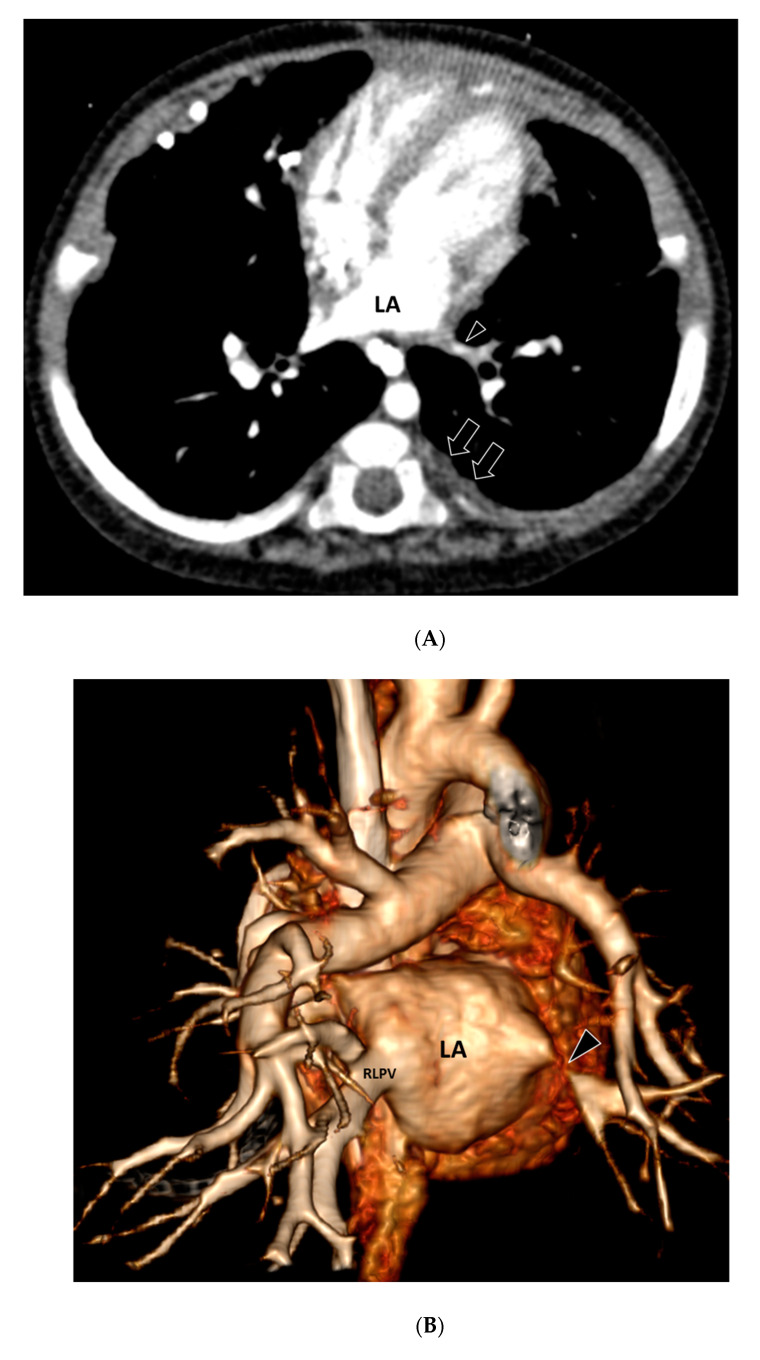

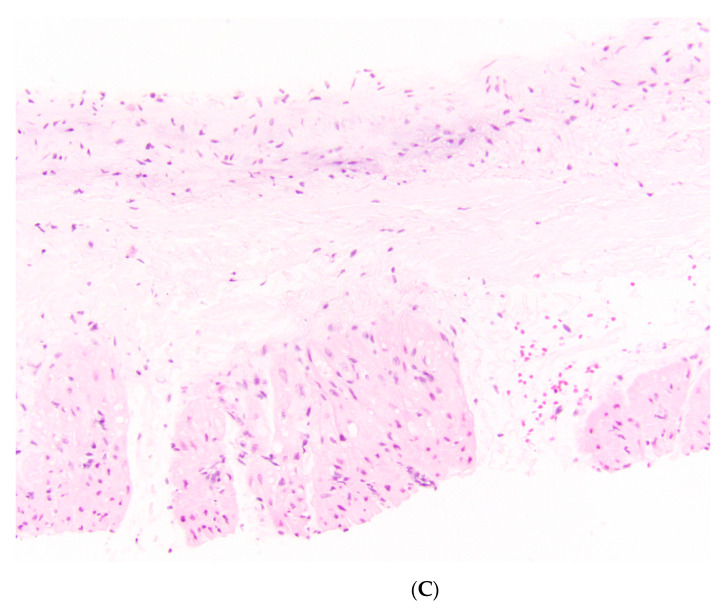
A 9-month-old male with complete atrioventricular canal defect and pulmonary vein stenosis who presented with hypoxemia and pulmonary hypertension. (**A**) Axial contrast-enhanced thoracic MDCT angiography CT image shows a markedly narrowed left inferior pulmonary vein (arrowhead). Pleural thickening (arrows) is also seen in the left posterior and medial pleura. Of note, unlike in Figure 1, no abnormal mediastinal soft tissue associated with PVS is identified. LA = Left atrium. (**B**) A 3-D volume-rendered vascular reconstruction CT image in the posterior projection view shows markedly narrowed left lower pulmonary vein (arrowhead). Patent right lower pulmonary vein (RLPV) is seen. LA = Left atrium. (**C**) Biopsy performed at an age of 8 months shows fibrointimal proliferation. Subjacent striated muscle is surrounded by paucicellular reactive-appearing fibroblastic cells (hematoxylin and eosin, original magnification, 200×).

Pleural abnormalities were seen in 16/20 (80%) thoracic MDCT angiography studies, including pleural thickening in 16/20 patients (80%) (Figure 1 and Figure 2), pleural effusion in 1/20 patients (5%), and pneumothorax in 1/20 patients (5%).

Mediastinal abnormalities were seen in 9/20 (45%) thoracic MDCT angiography studies, all of which were masses (*n* = 9; 45%) (Figure 1). All masses (9/9; 100%) were ill-defined, mildly heterogeneously enhancing, non-calcified soft tissue masses following the contour of PVS. There was no mediastinal lymphadenopathy.

### 3.5. Interobserver Agreement

The two reviewers agreed on all thoracic findings except on three occasions among the 20 included thoracic MDCT angiography studies. The discordance between the two reviewers was related to the presence of GGO in one patient, septal thickening in one patient, and effusion in one patient. The third reviewer, the tie-breaker, concluded that no GGO was present in the first discordant case, and that mild septal thickening was present in the second discordant case, and that small pleural effusion was present in the third discordant case. There was a high interobserver kappa agreement between the two independent reviewers for detecting extravascular abnormalities on thoracic MDCT angiography studies (k = 0.99).

## 4. Discussion

Our study, which specifically focused on the extravascular thoracic MDCT angiography findings of a unique pediatric patient cohort with isolated cardiac septal defect and PVS, sheds light on the characteristic extravascular thoracic MDCT angiography findings in children with PVS. The results of this study showed the characteristic extravascular thoracic MDCT angiography findings of GGO, septal thickening in the lungs and pleura in children with isolated cardiac septal defect and PVS. In addition, our study also found the presence of a novel, mildly heterogeneously enhancing, non-calcified soft tissue mass in the distribution of the PVS in the mediastinum. This important MDCT angiography imaging finding—a mediastinal soft tissue mass in the distribution of the PVS—is similar to a recently published finding in pediatric patients with primary PVS [13]. Therefore, this mediastinal soft tissue mass in the distribution of the PVS is most likely unique to pediatric patients with both primary (congenital) and acquired PVS.

**Figure 3:**
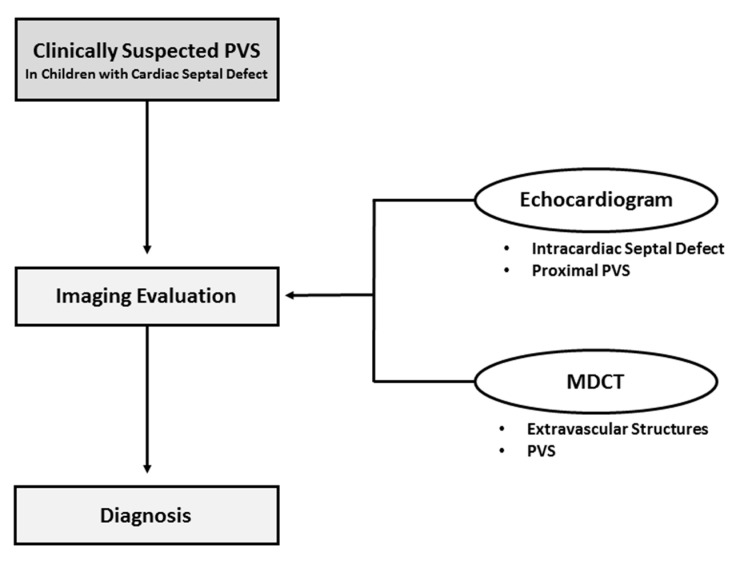
Recommended pathway to reach the correct diagnosis of pulmonary vein stenosis in children with cardiac septal defect PVS = Pulmonary vein stenosis MDCT = Multidetector computed tomography.

The majority of previously published radiological studies of the imaging assessment of PVS in both pediatric and adult populations have mainly focused on visualization of the vascular abnormality (i.e., narrowed pulmonary vein) for the initial diagnosis and follow-up evaluation after treatment [6,7,8,12]. By investigating the extravascular abnormalities in pediatric patients with PVS on thoracic MDCT angiography, our study was able to identify a unique mediastinal abnormality that may contribute to the development and progression of PVS in the pediatric population. For screening PVS in the pediatric population, we believe that both echocardiogram and MDCT play an important and complementary role (Figure 3). While an echocardiogram is useful for detecting intracardiac abnormalities (such as cardiac septal defect) and evaluating the caliber and patency of the proximal pulmonary veins, thoracic MDCT angiography can identify extravascular abnormalities, as well as evaluate the caliber and patency of the distal pulmonary veins that cannot be fully visualized or evaluated by echocardiography. Therefore, thoracic MDCT, which can provide a complete characterization of both vascular and extravascular abnormalities in pediatric patients with PVS, is a valuable non-invasive imaging modality for diagnosing PVS.

The most important finding of our study is the presence of a mildly heterogeneous enhancing, non-calcified soft tissue mass in the distribution of PVS in the mediastinum. It has been speculated that this abnormal mediastinal soft tissue mass may represent underlying myofibroblast-like soft tissue proliferation in pediatric patients with PVS [13,20,21]. In addition, previously published imaging studies have described similar findings in pediatric patients with complete pulmonary vein atresia [22,23,24]. Therefore, we postulate that perhaps this abnormal mediastinal soft tissue may be one of the underlying causative mechanisms for the narrowing of pulmonary veins in pediatric patients with PVS. However, we cannot exclude that it may represent an effect of PVS, postsurgical change, or simply a concomitant finding without a causal relationship. Interestingly, the frequency (45%) of this abnormal mediastinal soft tissue in our current study of pediatric patients with cardiac septal defect and PVS is substantially less than the frequency (93%) of a similar abnormal mediastinal soft tissue mass reported in primary pediatric PVS patients in a recently published study [13]. This difference in frequency raises the possibility that different pathophysiologic forces or perhaps an underlying congenital genetic propensity may play a role in the development of abnormal mediastinal soft tissue mass in pediatric patients with PVS. In addition, a recent publication also suggested that the left to right shunt itself and disturbed flow in the pulmonary veins may contribute to the development of PVS [25]. Future studies focusing on the direct correlation between the presence and degree of the left to right shunting and associated pulmonary venous flow disturbance with the development and degree of PVS will be beneficial. 

We believe that the results of our study are helpful for differentiating abnormal soft tissue associated with PVS in pediatric patients from other possible diagnostic etiologies of mediastinal masses in the pediatric population. The most common congenital mediastinal masses are foregut duplication cysts, including bronchogenic cysts, esophageal duplication cysts, and neurenteric cysts [26,27]. The imaging characteristics of congenital foregut duplication cysts, which are typically ovoid non-enhancing cystic masses, are substantially different from the solid, mildly enhancing, soft tissue mass seen in pediatric patients with PVS [26,27]. In addition, both primary neoplasms, such as lymphoma, and secondary neoplasms, such as metastatic lymphadenopathy, that occur in the mediastinum in children are usually well-defined, unlike the ill-defined abnormal mediastinal soft tissue following the pulmonary veins encountered in pediatric patients with PVS [26,27]. Lastly, infectious mediastinal lymphadenopathy from tuberculosis or histoplasmosis is often associated with concomitant calcification, which was not seen in the abnormal mediastinal soft tissue in pediatric patients with PVS [26,27]. We believe that clear knowledge of the constellation of characteristic imaging findings of mediastinal masses associated with PVS has a great potential for early and accurate diagnosis of PVS and differentiating it from other potential abnormalities in the pediatric population.

Other than the abnormal mediastinal mass seen in pediatric patients with PVS, our study found additional characteristic extravascular abnormalities on thoracic MDCT angiography studies, including GGO, septal thickening, and pleural thickening with frequencies of 90%, 45%, and 80%, respectively. Interestingly, these same three lung and pleural abnormalities (GGO (93%), septal thickening (33%), and pleural thickening (93%)) were also seen with similar frequency in a thoracic MDCT angiography study in pediatric patients with primary PVS [13]. We believe that these lung and pleural findings are secondary to the obstructed pulmonary veins. Therefore, the presence and frequency of these lung and pleural findings are similar in both primary and PVS. Specifically, we believe that the underlying pathophysiologic etiology of GGO, septal thickening, and pleural thickening is elevated pulmonary venous pressure in the interlobular septa and visceral pleural surface (due to obstruction of pulmonary venous return from narrowed pulmonary veins), resulting in imaging findings of GGO from alveolar (airspace) edema and septal thickening in the lungs, as well as pleural thickening in the pleura on thoracic MDCT angiography.

There are three main limitations to our study. First, although comparison of the results of our study (comprised of pediatric patients with cardiac septal defect and PVS) and the results of a previously published study (in pediatric patients with primary PVS) is informative, given the difference in patient characteristics (e.g., age and gender), future study with age- and gender-matched comparisons will be beneficial. Second, although our study population consisted of consecutive thoracic MDCT angiography studies with strict patient inclusion criteria, suboptimal quality thoracic MDCT angiography studies were excluded from the final study group. However, we would like to emphasize that the excluded thoracic MDCT angiography studies are only a small percentage (9%), and careful image quality assessment always strengthens the scientific value of investigation, particularly in studies with a small patient population mainly due to rare disorders. Lastly but also importantly, imaging studies were performed on five different MDCT scanners. However, we believe that this did not substantially affect the final results of our study because CT image thickness selection and types of reformatted images (i.e., coronal and sagittal reformation in both soft tissue and lung window settings) were the same for review.

In conclusion, our study is the first scientific investigation of the extravascular thoracic MDCT angiography abnormalities of pediatric patients with cardiac septal defect and PVS. We believe that the results of our study provide an important basis for comparison to the recently published study focusing on the extravascular thoracic MDCT angiography abnormalities of pediatric patients with primary PVS, as well as future studies of PVS with other underlying congenital and acquired causes. A clear understanding of the characteristic extravascular thoracic MDCT angiography findings (i.e., GGO, septal thickening, pleural thickening, and an abnormal mediastinal soft mass around the PVS) in the pediatric patients with cardiac septal defect and PVS has great potential for timely and accurate diagnosis, resulting in early treatment and optimum patient care.

## Figures and Tables

**Table 1:** Summary of Extravascular Thoracic MDCT Angiography Findings of Children with Cardiac Septal Defect and PVS.

Types of Extravascular Thoracic MDCT Angiography Findings	Number (Percentage) of Abnormalities (*N* = 20)
Lung Findings	
GGO	18/20 (90%)
Septal Thickening	9/20 (45%)
Nodule	0/20 (0%)
Mass	0/20 (0%)
Cyst	0/20 (0%)
Fibrosis	0/20 (0%)
Bronchiectasis	0/20 (0%)
Pleural Findings	
Pleural Thickening	16/20 (80%)
Pleural Effusion	1/20 (5%)
Pneumothorax	1/20 (5%)
Mediastinal Findings	
Mediastinal Mass	9/20 (45%)
Mediastinal Lymphadenopathy	0/20 (0%)

MDCT: Multidetector computed tomography. PVS: Pulmonary vein stenosis. N: Number. GGO: Ground-glass opacity.
